# *In Vivo* Toxicological Analysis of MnFe_2_O_4_@poly(*t*BGE-alt-PA) Composite as a Hybrid Nanomaterial for Possible Biomedical Use

**DOI:** 10.1021/acsabm.2c00983

**Published:** 2023-02-09

**Authors:** Rohit Kumar, Samir Bauri, Soumyamitra Sahu, Shaily Chauhan, Sunny Dholpuria, Janne Ruokolainen, Kavindra Kumar Kesari, Monalisa Mishra, Piyush Kumar Gupta

**Affiliations:** † Department of Life Sciences, Sharda School of Basic Sciences and Research, Sharda University, Greater Noida 201310 Uttar Pradesh, India; ‡ Department of Life Science, National Institute of Technology, Rourkela 769008 Odisha, India; § Department of Life Sciences, J.C. Bose University of Science and Technology, YMCA, Faridabad 121006 Haryana, India; ∥ Department of Bioproducts and Biosystems, School of Chemical Engineering, Aalto University, Espoo 00076, Finland; ⊥ Department of Applied Physics, School of Science, Aalto University, Espoo 00076, Finland; # Department of Biotechnology, Graphic Era Deemed to Be University, Dehradun 248002 Uttarakhand, India; ¶ Faculty of Health and Life Sciences, INTI International University, Nilai 71800, Malaysia

**Keywords:** nanocomposite, genotoxicity, biocompatible, Drosophila melanogaster, toxicity assessment

## Abstract

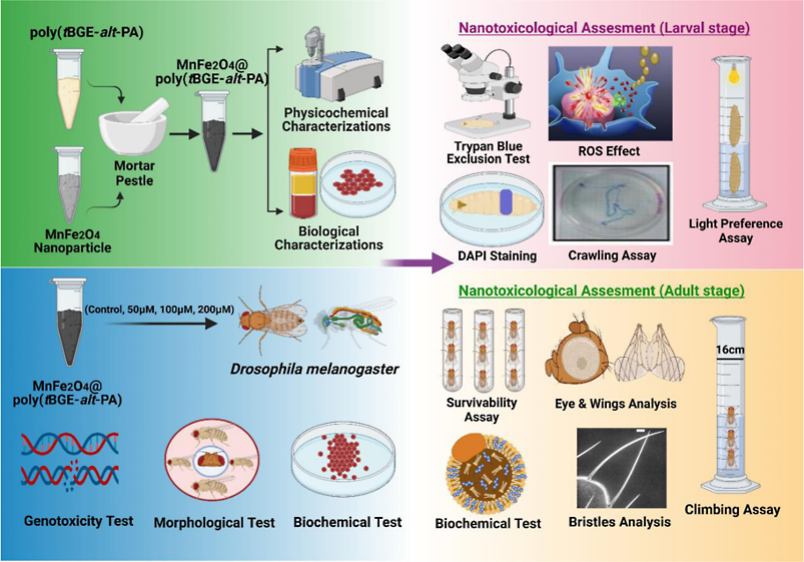

Nanocomposites have significantly contributed to biomedical science due to less aggregation behavior and enhanced physicochemical properties. This study synthesized a MnFe_2_O_4_@poly(*t*BGE-alt-PA) nanocomposite for the first time and physicochemically characterized it. The obtained hybrid nanomaterial was tested *in vivo* for its toxicological properties before use in drug delivery, tissue engineering fields, and environmental applications. The composite was biocompatible with mouse fibroblast cells and hemocompatible with 2% RBC suspension. This nanocomposite was tested on *Drosophila melanogaster* due to its small size, well-sequenced genome, and low cost of testing. The larvae’s crawling speed and direction were measured after feeding. No abnormal path and altered crawling pattern indicated the nonappearance of abnormal neurological disorder in the larva. The gut organ toxicity was further analyzed using DAPI and DCFH-DA dye to examine the structural anomalies. No apoptosis and necrosis were observed in the gut of the fruit fly. Next, adult flies were examined for phenotypic anomalies after their pupal phases emerged. No defects in the phenotypes, including the eye, wings, abdomen, and bristles, were found in our study. Based on these observations, the MnFe_2_O_4_@poly(*t*BGE-alt-PA) composite may be used for various biomedical and environmental applications.

## 1 Introduction

Metallopolymer nanocomposites are hybrid nanomaterials that combine metal component’s, electrical, optical, catalytic, and thermal properties with the polymer component’s flexibility, solubility, and manufacturability.^1^ These nanomaterials have been broadly utilized in biomedical applications due to their superior properties. Among the polymer components, polyesters have been used extensively in metallopolymer nanocomposite fabrication due to their better amphiphilicity, good biocompatibility, improved biodegradability, protection from UV radiation, and cellular enzymes.^2^ Furthermore, the metal component comprises metal/metal oxide nanoparticles like ZnO, Fe_3_O_4_, Fe_2_O_4_, CuO, MgO, ZnFe_2_O_4_, MnFe_2_O_4_, NiFe_2_O_4_, CoFe_2_O_4_, AgO_2_, and so on. These nanoparticles have been developed via different chemical and biological procedures. They exhibited significant role in the degradation of various inorganic/organic pollutants for environmental remediation and have been further utilized for therapeutic effects in various disease treatments.^3−5^

Further, *in vitro* cytotoxicity assays showed anti-cancer activity of biogenic MnFe_2_O_4_ nanoparticles against lung, breast, and skin cancer cells.^6^ Next, the polymer component of the metallopolymer nanocomposite also requires a cost-effective synthesis approach to reduce the usage of excess organic solvents and eliminate the postpurification step. Based on this, in a report,^7,8^ a low-molecular-weight metal-free semiaromatic alternating polyester [poly(*t*BGE-alt-PA) copolymer] was synthesized in one-pot step via anionic ring-opening copolymerization reaction.^7,8^ This copolymer was used to fabricate nanodrug carriers for combinatorial drug delivery of both doxorubicin and curcumin. Then, the rationale for developing such metallopolymer nanocomposites stands due to the slower degradation profile, lesser bioavailability, and agglomeration behavior of metal/metal oxide nanoparticles.^9^ By overcoming these physicochemical properties, metallopolymer nanocomposites have been used in drug delivery,^10^ biosensing, bioimaging, bioelectronics, and environmental remediation applications.^11^

Concerns regarding toxicological impacts on the human health and the environment have arisen in response to the expanding usage of nanoparticles worldwide in recent years. That is why it is always important to consider a nanomaterial’s potential risks and benefits before using it.^12^ Generally, the toxicity of newly developed nanoparticles/nanomaterials is majorly influenced by their physicochemical properties such as (1) the plasmonic properties, (2) coating on their surface, (3) particle size, (4) net surface charge, (5) shape/morphology, and finally (7) phase stability. Therefore, in the past few years, the nanotoxicology field has gained a lot of interest among material scientists, nanotechnologists, biomedical scientists, innovators, and entrepreneurs.^12^ However, two major significant factors have led to rapid progress in this area. The first factor is the large-scale production of nanomaterials with disturbing physical and chemical properties, and the second is the constant development of nanomaterials has stirred interdisciplinary research.^13,14^ For instance, nanomaterials have made massive progress in biomedicine. Nanomaterials have a large surface area-to-volume ratio, so they may display unpredictable interactions with cells and tissues. Several research studies have shown that nanomaterials exhibit highly complex interactions with cells and the environment.^12^ Many strategies to study the toxicity of nanomaterials are in progress. In the 20th century, it was shown that materials on a micrometer scale did not show any toxicity,^15^ but nanoscale materials might exhibit some toxic effects.^16,17^ The toxicity of nanomaterials can be studied in cell culture (*in vitro*) and in living organisms (*in vivo*) such as fish, flies, *Drosophila melanogaster*, mice, or rats.

In our previous experiments, we synthesized a bimetallic-semiaromatic polyester hybrid nanocomposite.^18^ The physicochemical properties of the synthesized MnFe_2_O_4_@poly(tBGE-alt-PA) hybrid nanocomposite were studied and reported previously. We later found that the nanocomposite was both biocompatible and hemocompatible in nature.^18^ In this work, we have evaluated the genotoxic and cytotoxic analysis of the newly developed metallo-polyester nanocomposites made up of MnFe_2_O_4_ nanoparticles and poly(tBGE-alt-PA) copolymers on the model organism *D. melanogaster*. For more than a century, scientists have used the fruit fly *D. melanogaster* as a laboratory organism to investigate a wide range of aspects of biology, such as heredity effects, aging, learning, behavior, and embryonic development.^19,20^ Developmental time points are mainly influenced by the environmental cycles, and reproduction ability may be changed. Disease-causing genes of *D. melanogaster* have almost 75% of homology with humans; that is why it is used as a genetic model to research various types of human diseases such as cancer, cardiovascular disease, and sleeping disease.^20,21^

## 2 Experimental Section

### 2.1 Synthesis and Characterization of MnFe_2_O_4_@poly(*t*BGE-alt-PA) Nanocomposite

MnFe_2_O_4_@poly(*t*BGE-alt-PA) nanocomposite was synthesized and reported in our previous study.^18^ In addition to the synthesis, we also characterized the physicochemical properties of the MnFe_2_O_4_@poly(*t*BGE-alt-PA) hybrid nanocomposite in our earlier paper.^18^ FTIR spectroscopy demonstrated successful hybrid nanocomposite synthesis. X-ray diffraction technology characterized the crystal structure of the hybrid nanocomposite. A thermogravimetric analysis instrument was used to investigate the thermal stability of the hybrid nanocomposite in a nitrogen environment.^18^ The surface topology of the hybrid nanocomposite was studied through field emission scanning electron microscopy.^18^ Using an MTT assay with mouse fibroblast cells (NIH3T3), the biocompatibility of the MnFe_2_O_4_@poly(*t*BGE-alt-PA) hybrid nanocomposite was evaluated.^18^ The hemocompatiblity of the MnFe2O4@poly(*t*BGE-alt-PA) hybrid nanocomposite was studied by the in vitro hemolysis test.^18^

### 2.2 In Vivo Toxicity Analysis on Fruit Fly Model

#### 2.2.1 Fly Strain and Culture Condition

All experiments were accomplished with the *Oregon R* strain of *D. melanogaster*. The flies were reared on standard fly food made of type I agar, sucrose (150 μM), corn meal, and yeast.^22,23^ Propionic acid and methyl-paraben were added to the food to protect them from fungal and microbial contamination.^22^ The flies were released to fresh food vials in a ratio of 5:3 (females and males, respectively). They were nurtured under optimum conditions of 60% relative humidity, 25 °C constant temperature, and 12 h day–night condition.^22,23^

At first, a stock solution of 2.5 mM concentration of MnFe_2_O_4_@poly(*t*BGE-alt-PA) was made by mixing the nanocomposite in powdered form in Mili Q water (7732185, SRL Chemicals, India), and the solution was stored at 4 °C. Fly food was prepared and divided into control and treatment. In the control food, no nanocomposite solution was added, whereas in the treatment food, different volumes of stock solutions were added to achieve various MnFe_2_O_4_@poly(*t*BGE-alt-PA) concentrations (50, 100, and 200 μM). Once the food was solidified, adult *Oregon-R* flies were freed to each vial. Larvae in their third instar of development and adult flies were employed extensively in the research.^22,23^ Adult flies fertilized with both control and treatment foods and laid eggs. The larvae were utilized in the experiment just 4 to 5 days after hatching.^24,25^

#### 2.2.2 Evaluation of Cytotoxicity and Genotoxicity of MnFe_2_O_4_@poly(*t*BGE-alt-PA) on Larval Gut

Larvae that had been fed MnFe2O4@poly(*t*BGE-alt-PA) were analyzed. Following the protocol of Priyadarshini et al. 2020, the larval gut was extracted and costained with dichloro-dihydro-fluorescein diacetate (DCFH-DA) (D6883, Sigma-Aldrich, Merck, Germany) and 4′,6-diamidino-2-phenylindole (DAPI) (D9542, Sigma-Aldrich, Merck, Germany).^22^ Cell nuclei are stained with DAPI (D9542, Sigma-Aldrich, Merck, Germany), whereas ROS (reactive oxygen species) produced by mitochondria was stained with DCFH-DA. Disintegrated nuclei were counted and represented against different concentrations of the nanocomposite fed to *Drosophila*. To determine the level of cellular stress caused by the nanocomposite treatment, a graph was also constructed showing the concentration against the intensity of DCFH-DA (D6883, Sigma-Aldrich, Merck, Germany). Following the protocol of Bag et al., we stained the intestines with trypan blue dye (93595, Sigma-Aldrich, Merck, Germany) to look for signs of membrane disruption caused by MnFe2O4@poly(*t*BGE-alt-PA) treatment.^26^

#### 2.2.3 Measurements of Oxidative Stress after MnFe_2_O_4_@poly(*t*BGE-alt-PA) Treatment on Larvae

Haemolymph collected from larvae in their third instar was utilized to measure oxidative stress. Briefly, 25 numbers of third instar larvae were collected. The larvae were cooled in a box and pricked near the thorax to stop melanization. Centrifugation of larvae was performed at 4 °C for 10 min at 4500 rpm (Eppendorf-centrifugation 5430/5430R, Germany). 5 μL of hemolymph was taken in an Eppendorf tube of 1.5 mL, and 10 μL of 1X phosphate-buffered saline (PBS) was added to the tube. An equal volume of 1.6 mM nitroblue tetrazolium (NBT) solution (11383213001, Sigma-Aldrich, Merck, Germany) was added to the mixture and left for 1 h in the dark. NBT (11383213001, Sigma-Aldrich, Merck, Germany) assay was performed on the hemolymph according to the protocols of Nayak et al. 2020 and Bag et al. 2020.^26,27^ NBT (11383213001, Sigma-Aldrich, Merck, Germany) (1.6 M) solution was given to the hemolymph and left for 1 h in the dark. The reaction was stopped after 1 h by adding an equivalent amount of 100% glacial acetic acid (GAA) (A6283, Sigma-Aldrich, Merck, Germany) and incubating for 5 min. Then, 150 μL of 50% GAA (A6283, Sigma-Aldrich, Merck, Germany) was mixed, and 200 μL of the solution was poured in the well of a 96-well plate, and the absorbance was taken at 595 nm with the help of a microplate reader (Elisa Biobase, EL10A).

#### 2.2.4 Larvae Crawling Behavior Assay

Larval movement patterns show how neuronal damage is caused by hazardous chemicals and various materials used for toxicology analysis on *D. melanogaster*. Larvae have a characteristic rhythmic crawling movement; they travel in a straight line at a constant rate most of the time. Changes in how one crawls are a significant sign of neural malformation. The crawling assay was done with five third instar larvae from each treatment concentration (50, 100, and 200 μM) of MnFe_2_O_4_@poly(*t*BGE-alt-PA) nanocomposite and control.^28^ Larvae were isolated from the food and washed in 1X PBS to clear the food particles. 2% agar-containing Petri plates were made as the crawling surface.^29^ Initially, the larvae were put on an agar plate to adapt to that environment. One by one, the larvae were picked to the center of a different agar plate and placed on a graph paper to observe their crawling path. In the meantime, the video was recorded (Canon EOS 3000D, Japan). The time taken by each larva to reach the periphery of the Petri plate was measured, and that time was divided by 1 min to calculate the crawling speed. On the agar gel, the larvae left a trailing impression of their crawling path. Markers were used to sketch the larvae’s crawling routes, and their average speed per second was then plotted.

#### 2.2.5 Trypan Blue Staining

Third instar larvae were stained with Trypan blue (93595, Sigma-Aldrich, Merck, Germany) following a reported protocol.^22^ We placed 10 larvae from each group (control and each treatment) into a 0.5 mL centrifuge tube. Before the experiment, the larvae were collected and washed thoroughly in PBS (1 X) to eliminate any leftover feeding particles. All larvae were submerged into the trypan blue (93595, Sigma-Aldrich, Merck, Germany) and placed in a dark place for 45 min at room temperature (RT). After 45 min, the larvae were washed in PBS solution to remove any trace of color consumed or left on their surface. After imaging the larvae using a stereomicroscope (ACCU-SCOPE Inc., Commack, New York), we looked for signs of cell damage.^22,30^

#### 2.2.6 Touch Sensitivity Study

Various organs, including the nervous system, different body parts, and neuromuscular junctions, work together to produce touch. Central pattern generators (CPGs), located in the brain, are the source of stimuli. Even without outside sensory input, the oscillatory network continues to move. However, without a peripheral nervous system (PNS) stimulus loop, the body’s segmentation expands and contracts uncoordinatedly. Signaling from the CPG that initiates peristaltic movement begins in the late embryonic stage and persists throughout the larval stage. The chordotonal organ of the PNS receives a signal for sensing and movement from the CPG.^31,32^ Any sensory impairment thus impairs the larvae’s ability to respond to stimuli. Larval behavior is examined, and the neural defect can be scored. The exact path has been followed for isolating the larva, washing, and acclimatization in the agar plate environment. The thoracic region of the larva was gently pricked with an eyelash glued to a toothpick which acts as mechanical stimuli. The responses of the larvae were noted and scored according to Dhar et al. 2020.^29^

#### 2.2.7 Climbing Assay

Climbing is an innate behavior of *Drosophila. Drosophila* always tries to climb vertically against gravity, so they showed negative geotactic behavior. Climbing reflects the neurodegeneration in the *Drosophila* model. Adult fruit flies’ locomotory behavior was evaluated using this same technique as in a reported protocol.^22^ 3-day old flies (30 adult flies) were moved to the climbing apparatus from three distinct concentrations.^33,34^ Flies were taped gently to the bottom of the vial, and the duration of 10 s to climb 16 cm of the tube was recorded. All concentrations of the nanocomposite and controls were tested five times using this methodology. Percentages of total flies were used to determine the number of flies in each group that successfully climbed the mark of 16 cm in the time of 10 s.^35^

#### 2.2.8 Survivability Study

Toxicology was evaluated using the same method described by Ales Panacek et al. 2011. Flies were fed with various concentrations of (50, 100, and 200 μM) MnFe_2_O_4_@poly(*t*BGE-alt-PA), including control, and they laid their eggs on the food. However, the results vary depending on the concentration of the nanocomposite. Each vial was labeled with a symbol for a developing fly egg, and daily counts of the hatched flies were recorded. The proportion of surviving flies in each concentration was used to create the graph.^36,37^

#### 2.2.9 Average Body Weight Analysis

Thirty adult flies (15 males + 15 females) were sampled from each concentration shortly after hatching, and their weight was compared with that of the control group.^22^

#### 2.2.10 Larval Light Preference Assay

This experiment detected an early photoreceptor deficiency using the approach described by Sabat et al. 2016.^38^ A Petri dish was divided into four quadrants, with the opposite quadrant being colored black (two quadrants are black). Then, 1% agarose was added and let to set. Fifteen third instar larvae from both the control and treatment vials were kept in the dark for 6 h before the experiment began. The larvae were placed on the agar plate, and the lid with the same marking as the Petri plate was closed. The Petri dish was illuminated uniformly, and the larvae were given 5 min to move freely between the dark and light sections. After 5 min, we removed the lid and tallied the larvae in each section. Each batch of larvae performed the test three times, and the experiment was conducted in three sets.^22,38,39^

#### 2.2.11 SEM and EDS Scanning

Three guts were separated from the third instar larva of each concentration and stored at 4 °C in 4% paraformaldehyde (PFA) (158127, Sigma-Aldrich, Merck, Germany). To eliminate the extra PFA (158127, Sigma-Aldrich, Merck, Germany), the guts were rinsed with PBS. The guts were dehydrated using a graded serial dehydration method that involved increasing the percentage of ethyl alcohol (1.00983, Sigma-Aldrich, Merck, Germany). The concentrations of the ethyl alcohol used were 30, 50, 70, 90, and 100%. The desiccated guts were mounted on a slide containing carbon tape and then punctured in the midgut region to expose the gut lumen. Scanning electron microscopy (SEM) (JEOL JSM-6480LV) analysis followed by coating the samples with platinum. The quantity of manganese and iron was determined by energy-dispersive spectroscopy (EDS) analysis.^40,41^

#### 2.2.12 Phenotype Observation

The nanomaterial’s character was examined by checking phenotypes to determine whether it benefits or harms the model organisms. Fifty adult flies were screened for phenotypes in their eyes, wings, bristles, and abdomens. The images were taken with a stereo microscope (Motic SMZ-171).^42,43^ The adult phenotypic analysis showed no defects in the eyes, wings, bristles, or abdomens.

### 2.3 Statistical Analysis

With the help of a software GraphPad Prism 9.0, we analyzed all experimental data. Using the significance *P < 0.05, **P < 0.01, and ***P < 0.001 from unpaired two-tailed student t-test, the data were interpreted with the mean ± SEM values.

## 3 Results

### 3.1 Synthesis and Physicochemical Characterization of MnFe_2_O_4_@poly(*t*BGE-alt-PA) Nanocomposite

The MnFe_2_O_4_@poly(*t*BGE-alt-PA) hybrid nanocomposite was fabricated and studied for the first time in our prior work.^18^ No chemical interactions were seen between the copolymer and the produced hybrid nanocomposite; it was found to be crystalline and thermostable.^18^ MnFe_2_O_4_@poly(*t*BGE-alt-PA) hybrid nanocomposite has a net negative charge on their outermost layer. Further, the nanocomposite was hemocompatible and biocompatible.^18^

### 3.2 Cytotoxicity and Genotoxicity Analyses of MnFe_2_O_4_@poly(*t*BGE-alt-PA) on Larval Gut

**Figure 1:**
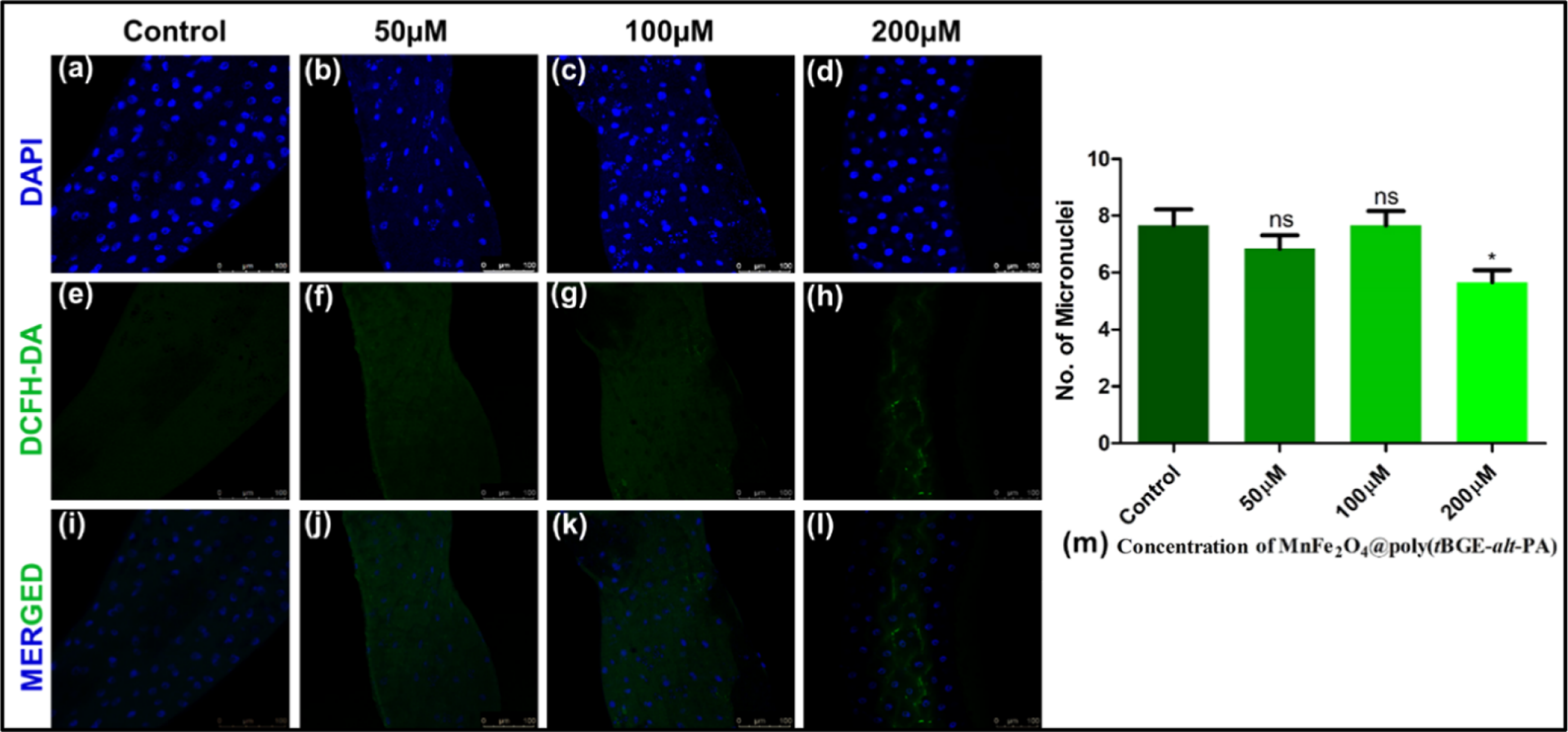
DAPI and DCFH-DA staining of the larval gut. (a-l) Representation of the number of micronuclei formation due to DNA breakdown of control and treatment groups with the help of micronuclei counting. (m) No significant DNA damage was observed in 50 and 100 μM of concentrations, although it was measured significant damage at higher concentration (200 μM) of nanocomposites treatment.

The flies were fed with the nanocomposite, which passed through the intestine and interacted directly with the epithelial cells of the intestine. The toxicity of the cells was examined to scrutinize whether the nanocomposite induced any damage to the cells. DAPI (D9542, Sigma-Aldrich, Merck, Germany) was used to identify deoxyribonucleic acid (DNA) damage after exposure of the cells to any toxic compound. The DNA damage observed was insignificant in the nanocomposite treatment shown in Figure 1. The graph shows the number of micronuclei formation due to DNA breakdowns in the treated groups (Figure 1). After internal staining of the larval gut by DCFH-DA (D6883, Sigma-Aldrich, Merck, Germany), it was observed that the MnFe_2_O_4_@poly(*t*BGE-alt-PA) nanocomposite could reduce the internal ROS activities shown in Figure 1. This finding was supported by the NBT (11383213001, Sigma-Aldrich, Merck, Germany) assay, suggesting that the nanocomposite helps reduce ROS and protects the cell from oxidative stress.^34,44^

### 3.3 ROS Analysis

**Figure 2:**
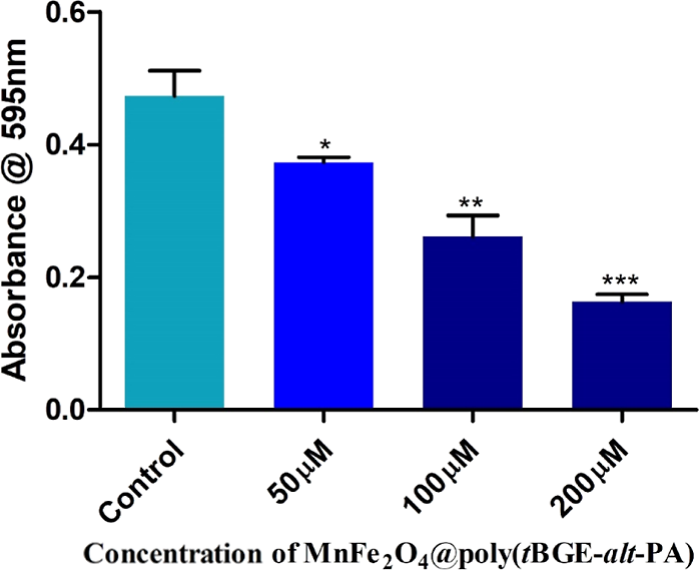
The mean interaction plot of the NBT assay of larvae shows that increases in the concentration of the MnFe_2_O_4_@poly(*t*BGE-alt-PA) nanocomposite play a vital role in reducing the ROS and protect the cell from oxidative stress.

An NBT (11383213001, Sigma-Aldrich, Merck, Germany) assay was performed in the third instar larval hemolymph to measure the amount of intracellular ROS from the NBT (11383213001, Sigma-Aldrich, Merck, Germany) assay. ROS formation reduced significantly in the nanocomposite-treated group compared to the control. Thus, the NBT (11383213001, Sigma-Aldrich, Merck, Germany) assay suggests that the MnFe_2_O_4_@poly(*t*BGE-alt-PA) nanocomposite plays a vital role in ROS scavenging^41,45^ as shown in Figure 2. In control, the absorbance value at 595 nm was found to be 0.4731 ± 0.38. In 50 μM concentration, the value decreased to 0.3733 ± 0.007. In 100 μM concentration, the absorbance was further decreased to 0.2611 ± 0.032, and for 200 μM concentration, the absorbance was 0.1631 ± 0.011. The absorbance of the NBT (11383213001, Sigma-Aldrich, Merck, Germany) assay is directly proportional to the quantity of ROS generated, which ultimately correlates with the level of oxidative damage to the cells.^46^ The amount of ROS reduction that occurs compared to the control is represented in the graph (Figure 2).

### 3.4 Crawling Assay

**Figure 3:**
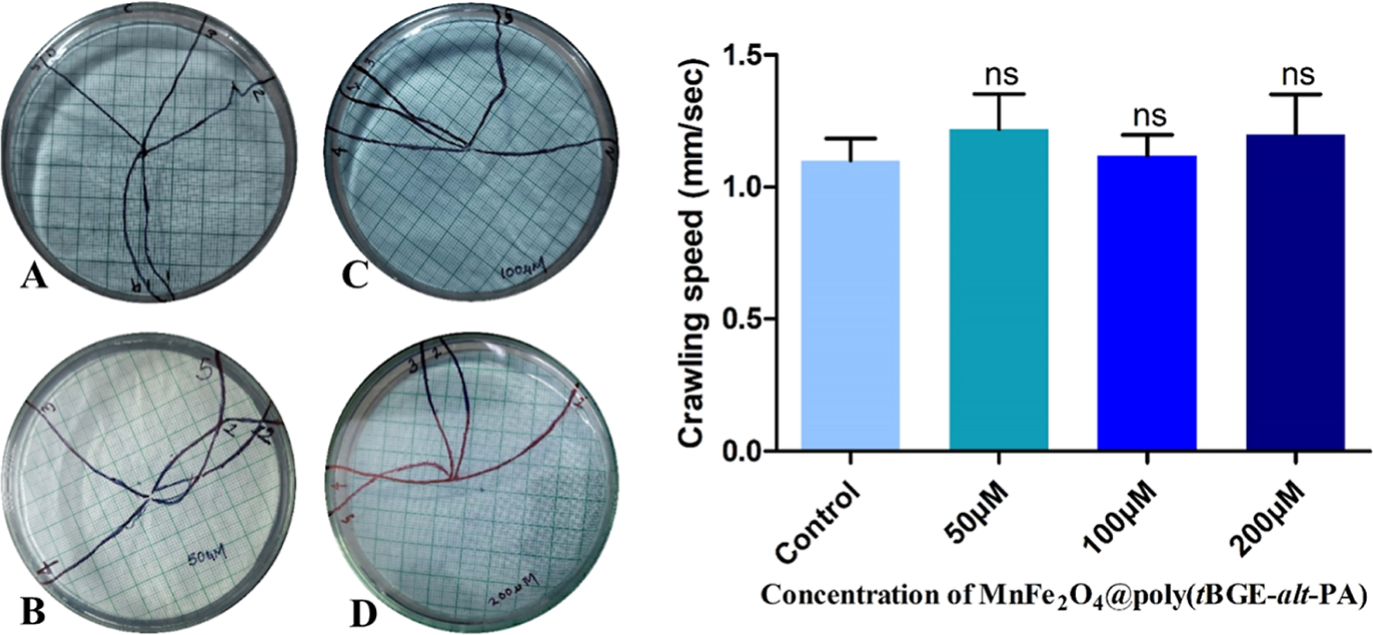
Genotoxicity studies in the larvae of *Drosophila melanogaster*. The mean interaction plot of the larval crawling speed of (A) Control and (B,C, and D) 50, 100, and 200 μM, respectively, in all treated groups. Larval crawling path and speed did not show significant changes in various treated concentrations comparable to control larval groups.

The crawling behavioral test is a more practical assay to explore the neuronal abnormalities in an early stage of larva for the neuronal mechanosensory investigation. The crawling behavior of third instar larvae was studied in the Drosophila model. The neuronal toxicity caused by the NP exposure can disrupt the coordinated crawling of larvae. The healthy larvae move in a straight line, whereas the abnormal ones zigzag and sometimes slow down. Thus, the crawling assay in larvae is preferable for identifying abnormalities in gene expression that might result in fatalities during the pupal and adult stages. In the crawling assay, no distinct curve or turn has been recorded for the control larvae. There was no significant crawling path change for the treatment concentrations of 50, 100, and 200 μM. In the control vial of larvae, 1.101 ± 0.082% were able to cover the distance in mm/s, whereas 1.219 ± 0.133% were able to cover the distance in 50 μM, 1.119 ± 0.078% in 100 μM, and 1.199 ± 0.151% in 200 μM. The crawling speed of third instar larvae shows that all treated larva significantly covers the same distance in mm/s comparable to control larvae. The larvae tracking paths and the crawling speed plot is demonstrated in Figure 3.

### 3.5 Living and Nonliving Cell Analysis

**Figure 4:**
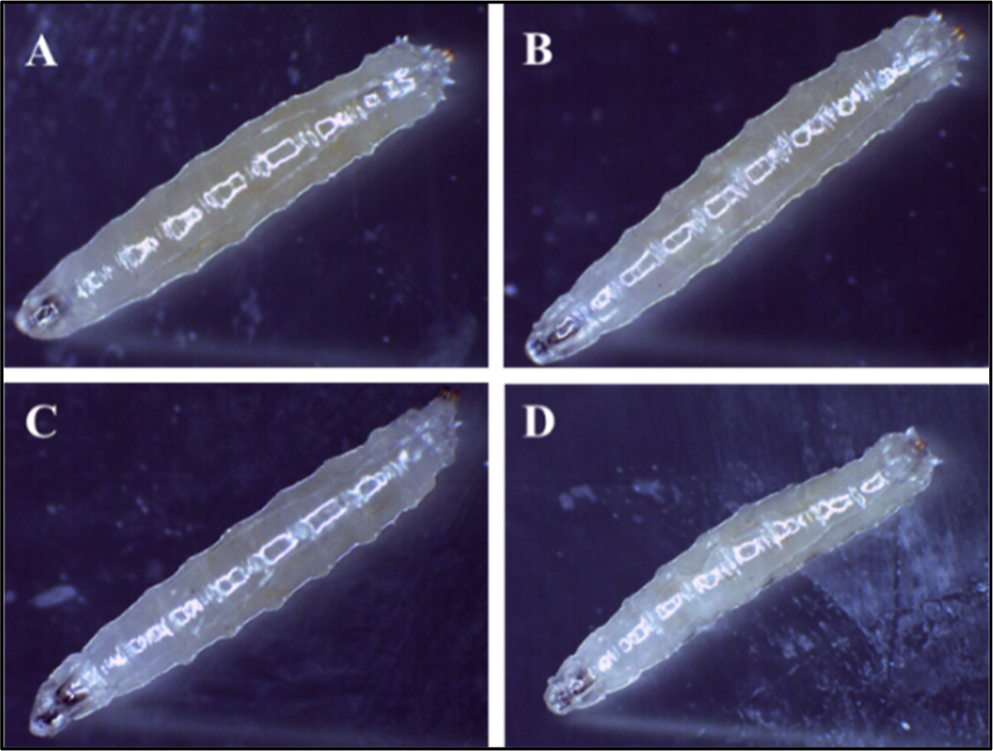
Trypan blue exclusion assay executed in third instar larvae and the effect of the nanocomposite in the third instar larval stage (trypan blue, used as a marker for dead cells, also detects the presence of any tissue damage). (A) Larvae from the control show no sign of cell or tissue damage. Also, (B) 50, (C)100, and (D)200 μM nanocomposite-treated larvae did not show any internal gut damage.

The use of trypan blue (93595, Sigma-Aldrich, Merck, Germany) staining allows for the differentiation of living cells from nonliving ones. Positive trypan blue (93595, Sigma-Aldrich, Merck, Germany) staining is not observed in any concentration of the nanocomposite that was given to the food vial, indicating nondamage to the gut’s inner layer.^47^ Even at higher concentrations of MnFe_2_O_4_@poly(*t*BGE-alt-PA), 200 μM, no significant toxicity was seen in the *Drosophila’s* larval stages, which are in a voracious feeding stage as shown in Figure 4.^48^

### 3.6 Climbing Assay

**Figure 5:**
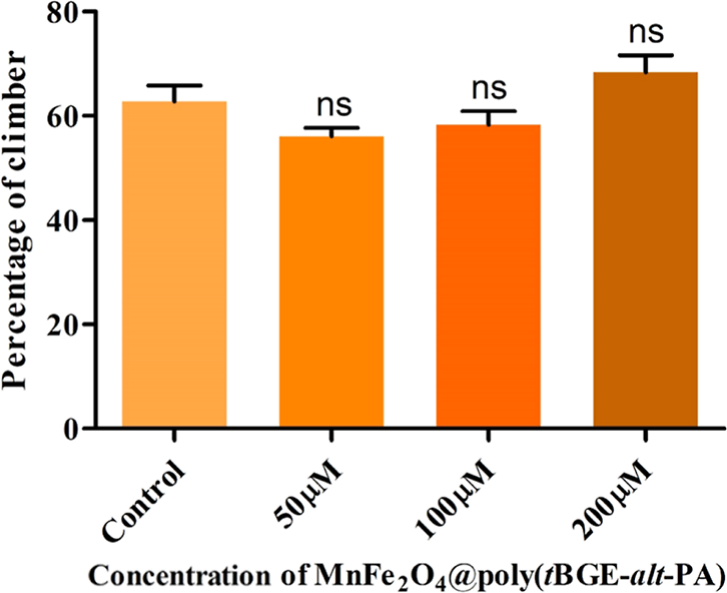
Climbing assay in each nanocomposite treated setup comparable with control flies; behavioral abnormality was not significantly found with increases up to 200 μM concentration.

The climbing experiment describes the behavioral changes that occur in flies in response to gravity. The number of flies that could ascend to 16 cm in the 10 s is used to analyze this test. In due order, the number of flies that could climb up to 16 cm was normalized to 100%. The assay was performed six times (*N* = 6) for each concentration, including control. In the control flies vial, 62.78 ± 3.03% were able to climb, whereas 56.11 ± 1.59% in 50 μM, 58.33 ± 2.55% in 100 μM, and 68.33 ± 3.31% in 200 μM were able to climb up to the 16 cm mark. The result of the climbing assay is plotted in a graph shown in Figure 5. The climbing ability was not significantly changed in the treated flies compared to the control flies of the setup.^22,49^

### 3.7 Survivability Study

**Figure 6:**
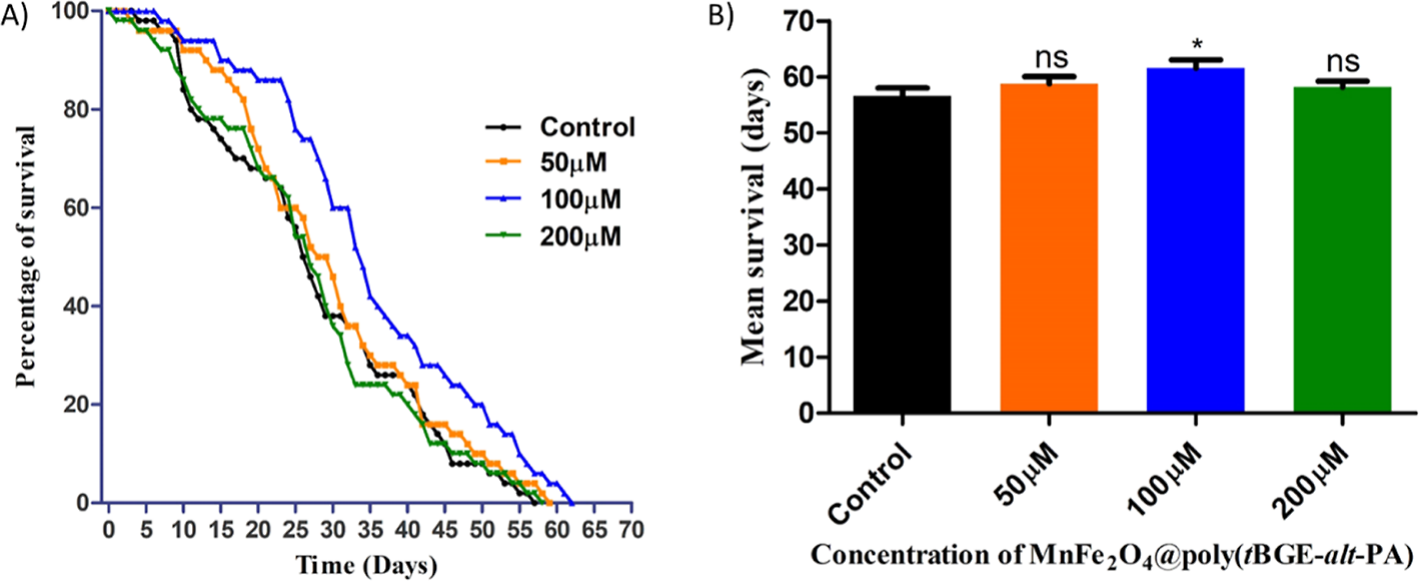
Survivability assay. (A) Percentage survival. (B) Mean survival for flies fed on standard and nanocomposite mixed food. No significant survivability differences found in control, 50, and 200 μM concentrations of the nanocomposite, whereas the 100 μM concentration of nanocomposite increases the survivability of flies.

This assay determines the number of survival days of flies that successfully undergo eclosion from the pupal stages. Fifty freshly emerging flies (25 males and 25 females/vial and five vials/group) were put to standard food with or without nanocomposite treatment (50, 100, and 200 μM) and control. On alternating days, the surviving flies were switched to fresh food containing MnFe_2_O_4_@poly(*t*BGE-alt-PA) and control (without nanocomposite treatment). Dead flies were counted every day until the final fly perished. There were no significant differences found in control, 50, and 200 μM concentrations [56.60 ± 1.44, 58.80 ± 1.28, and 58.20 ± 1.02 days (*N* = 5)], whereas the 100 μM concentration increased the survivability of hatched flies to 61.60 ± 1.44 days (*N* = 5), as shown in Figure 6A,B).^36,37,50^

### 3.8 Adult’s Average Body Weight Analysis

**Figure 7:**
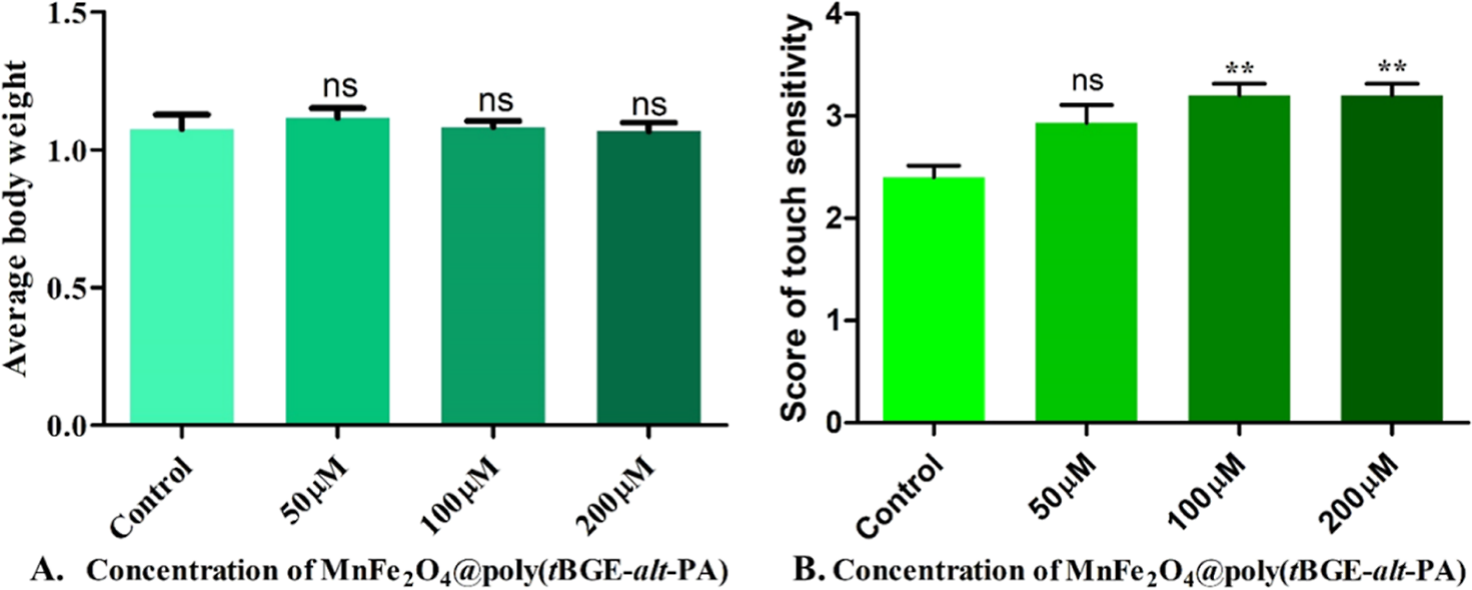
(A) Weight of adult flies: 30 treated and control flies were weighed (15 males and 15 females). The average weight of a single fly in each group is plotted to its corresponding concentration and compared with the control. (B) Graph showing touch sensitivity score. Increasing the nanocomposite concentration up to 200 μM affect larval reflexes or neuronal development. A significant (**) differences were observed in touch sensitive score of treated flies at 100 μM and 200 μM as compared to control.

The weight of adult flies was determined from several treatment and control vials to assess body growth and size. Thirty treated and control flies were weighed (15 males and 15 females). Then, the average weight of a single fly in each group was calculated and found to be 1.076 ± 0.052 mg in the control group, whereas in 50 μM treated concentration, it was 1.117 ± 0.036 mg. Likewise, the weight of a single fly in 100 μM treated concentration was 1.083 ± 0.022 mg. For 200 μM, the value was 1.068 ± 0.032 mg. In the body weight of the adult fly, no remarkable difference or defect has been found in the treated and the control groups. Treatment concentrations of MnFe_2_O_4_@poly(*t*BGE-alt-PA) (50, 100, and 200 μM) did not show a decrease in the average weight of the fly at various concentrations. A graph was plotted to represent the body weight, as shown in Figure 7A.^22,51^

### 3.9 Touch Sensitivity Test

A sensation of touch is another fundamental activity of animals, with implications for anything from learning about their surroundings to interacting with others. No mechanoreceptor potential C (NOMPC) is a subset of the MYOC gene family that mediates mechanical stimuli into electrical signals,^46^ making it a key player in the process of feeling touch.^28,52^ In our experiment, we found that at 50 μM concentration of nanocomposite treatment, the larvae’s touch sensitivity score was 2.93 ± 0.18, which was practically identical to the control group’s score that was 2.40 ± 0.12 (the scores for both groups were between 2 and 3, indicating that the larvae hold their movement before moving forward). Similarly, at 100 and 200 μM concentrations, the touch sensitivity score was 3.20 ± 0.12 for both the cases (the scores for both groups were more than 3 but below 4, indicating that the larvae turned 90^°^ and then moved), as shown in Figure 7B.^29^ Increasing the nanocomposite concentration up to 200 μM did affect larval reflexes or neuronal development.

### 3.10 Larval Light/Dark Preference Assay

**Figure 8:**
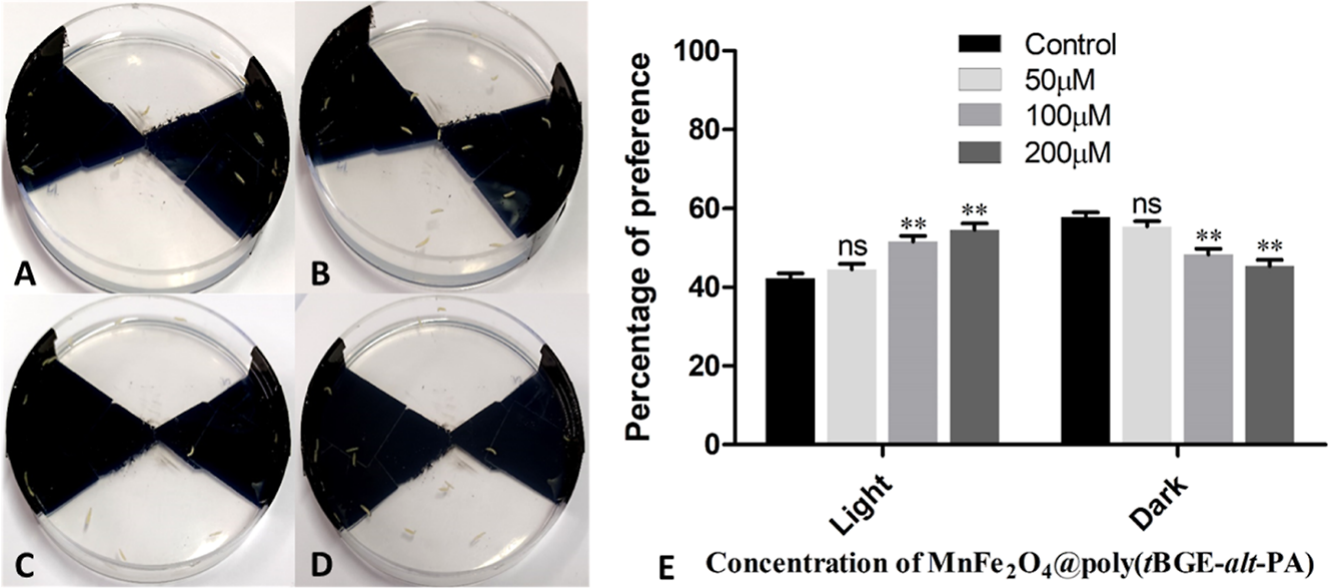
Light/dark preference assay of larva, Petri dishes. (A). Control and (B–D). 50, 100, and 200 μM doses of the hybrid nanocomposite-treated larval groups, respectively, (E). Graph of the light/dark preference test (*N* = 12 (180 larvae) per time point).

The larva’s light preference test was done to look for any early defects in the light-sensing neurons. In this experiment, the percentage of larvae attracted to light increased as the concentration of nanocomposite treatment increased. The control group’s percentage of larvae attracted to light was 42.22 ± 1.29%. There were 44.56 ± 1.39% of light-sensitive larvae in 50 μM, 51.62 ± 1.38% in 100 μM, 54.60 ± 1.60% in 200 μM, as shown in Figure 8.^25,39^ However, light was avoided or dark was preferred by 57 ± 1.29% larvae from the control group, 55.44 ± 1.39% in 50 μM, 48.38 ± 1.38, and 45.40 ± 1.60% in the case of 200 μM hybrid nanocomposite-treated larval groups.^53^

### 3.11 Analysis of the Presence of Elements in the Larval Midgut by SEM-EDS

**Figure 9:**
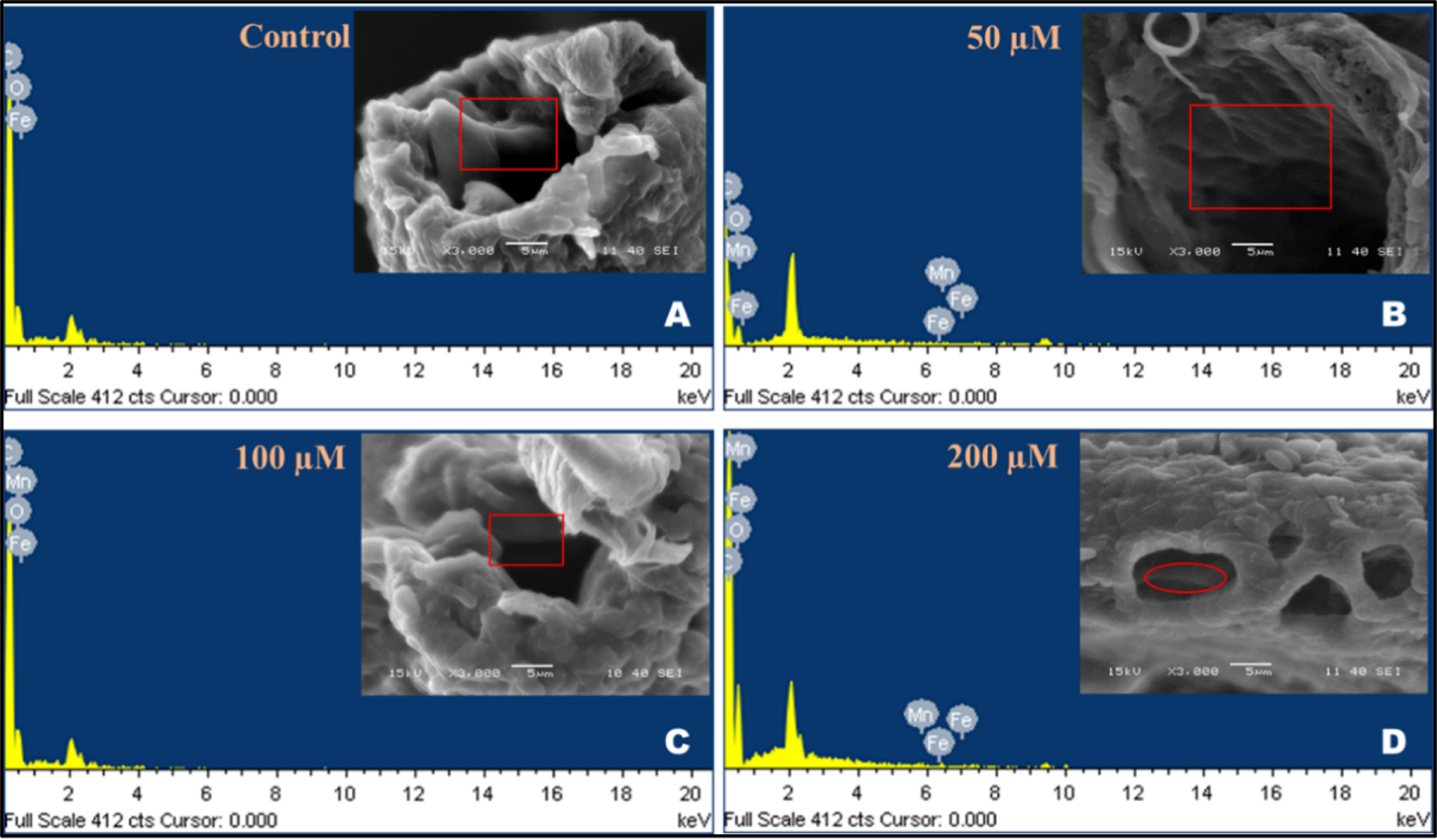
Elemental analysis of the larval gut indicates nanocomposite deposition within the gut of treated larvae (B, C, and D). In contrast, no nanocomposite traces were found in the gut of the control (A).

To verify the larva’s consumption of MnFe_2_O_4_@poly(*t*BGE-alt-PA), the gut of the third instar larva was examined using SEM/EDS. Each experimental setup’s larval midgut was taken out and analyzed for elementary deposition using SEM (JEOL JSM-6480LV), as shown in Figure 9A–D. The EDS findings verified that the MnFe_2_O_4_@poly(*t*BGE-alt-PA)-treated larval gut had a larger proportion of manganese and iron deposition than the untreated control gut. There was very less amount of iron and no traces of manganese found in the gut of untreated larvae. However, the percentage of manganese and iron increased according to the increasing concentration of MnFe_2_O_4_@poly(*t*BGE-alt-PA) treatment. This finding supports that no toxicity is induced by the nanocomposite and confirms the deposition of the hybrid nanocomposite in the gut.^54^

### 3.12 Phenotype Observation

**Figure 10:**
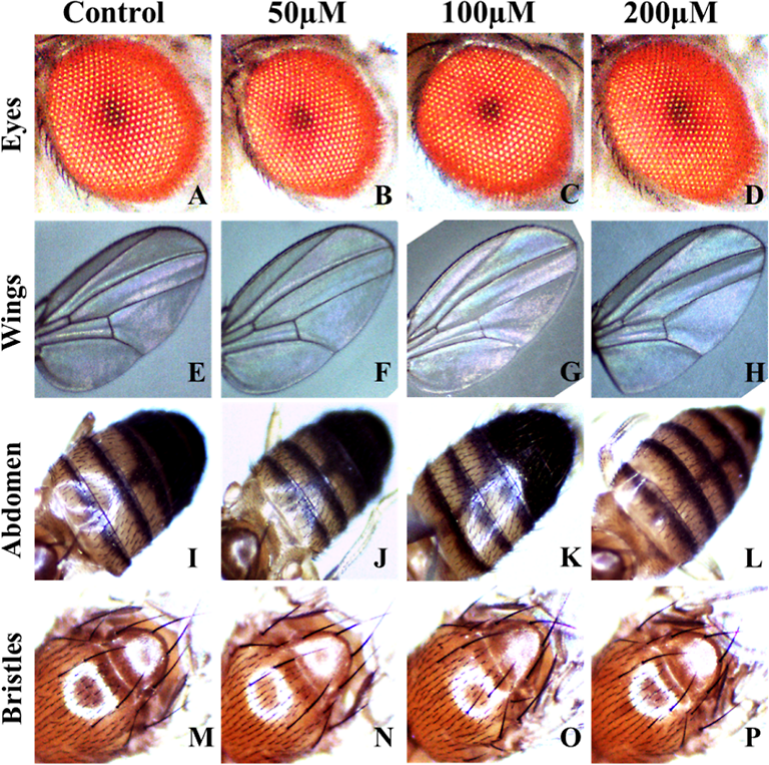
Eyes, wings, abdomen, bristles phenotype. In control (A,E,I,M) and treated concentrations of 50 μM (B,F,J,N), 100 μM (C,G,K,O), and 200 μM (D,H,L,P), no remarkable difference or defect has been found in first-generation *D. melanogaster*.

Wing venation pattern, eye coloring, thorax bristle count, and abdomen structure were all monitored to see if there were any phenotypic alterations due to the treatment with the nanocomposite. No remarkable difference or defect in the first-generation *D. melanogaster* treated and control groups is shown in Figure 10.

## 4 Discussion

A major cause for concern is the increasing number of biological uses for nanomaterials, which might present significant risks to human health.^55^ Several studies have demonstrated that nanomaterials’ size, shape, and structure significantly alter the actions and responses of living organisms.^56^ Due to the sequence completion of the human and *Drosophila* genomes, many loss-of-function experiments have been streamlined, providing us with a fundamental understanding of the genes involved in many diseases. Drug development may be advantageous due to the *in vivo* model organism, *D. melanogaster*, and a smaller gene family since fewer genes need to be controlled to produce acute circumstances for drug screening.^57^ Moreover, other NPs, such as ferrous, manganese, zinc oxide, and silica, influence the neurons by inhibiting neuronal function via dopamine depletion, increasing heat stress, and causing edema development. Increases in dosage, concentration, and particle size affect somatosensory neurons in the dorsal root ganglia.^58^ In the current study, the synthesized and physicochemically characterized nanocomposite was administered through the food immediately after hatching from the eggs, and the larva started feeding treated food. After being ingested, this nanocomposite did not alter larval behavior or produce any abnormal phenotypes. When fed with the nanocomposite, the larvae exhibited no signs of neural dysfunction. In this investigation, no larval fatality or aberrant behaviors were identified; nevertheless, if larvae struggle to survive the stress during larval stages, the deficiency appears to be seen in the adult stage within the disrupted developmental process during metamorphosis.

The larval crawling experiment is used as a method for evaluating neuronal activity. Experimental studies on crawling larvae show that increasing MnFe2O4@poly(*t*BGE-alt-PA) concentrations did not affect their crawling speed. The crawling assay shows that increasing MnFe2O4@poly(*t*BGE-alt-PA) concentrations did not affect their crawling.^28^ The larvae treated with MnFe2O4@poly(*t*BGE-alt-PA) showed no signs of confusion, as shown by the lack of abrupt turns or decreased crawling speed in their tracks. Trypan blue staining was carried out to analyze the extent of the cellular damage. Trypan blue staining indicates the number of dead cells in the digestive tract after treatment with several concentrations of MnFe2O4@poly(*t*BGE-alt-PA).^22,30^ The *Drosophila’s* larval stages, which are at a voracious feeding stage, showed no signs of toxicity even when exposed to MnFe2O4@poly(*t*BGE-alt-PA) at concentrations as high as 200 μM.

The larval intestine was examined using a scanning electron microscope to see if MnFe2O4@poly(*t*BGE-alt-PA) had any impact on cellular pathways or to check the nanocomposite deposition within the gut of treated larva. The EDS findings substantiated that the MnFe2O4@poly(*t*BGE-alt-PA)-treated larval gut had a larger proportion of manganese and iron deposition than the untreated control gut. The EDS results confirmed an increase in manganese and iron deposition in the MnFe2O4@poly(*t*BGE-alt-PA)-treated larval gut compared to the control gut. However, as the MnFe2O4@poly(*t*BGE-alt-PA) concentration was increased, the amount of manganese and iron also increased in the gut. These results further support that the nanocomposite does not cause any toxicity, and they verify the deposit of the hybrid nanocomposite in the gut.^54^ Oxidative stress is generated by the increased production of ROS carried by silver, gold, and titanium nanoparticles. One well-established technique for measuring ROS concentration is the NBT test.^47^ NBT was done to quantify the ROS in the treated larva compared with the control one. The absorbance was taken using a microplate reader, and the graph also measured the difference.^46^ When comparing the highest and lowest concentrations of MnFe2O4@poly(*t*BGE-alt-PA), the amount of oxidative stress produced at the lowest concentration, 50 μM, and the highest concentration, 200 μM, was considered negligible. Different phases of *Drosophila* development, characterized by crawling assay, climbing assay in the larvae, and phenotypic analysis, including the eye, wings, abdomen, and bristles, suggest that the MnFe_2_O_4_@poly(*t*BGE-alt-PA) composite has no such cytotoxicity as well as genotoxicity. The findings of this study show that our experimental design may be employed as a secure injestion system for toxicological research on various *in vivo* model organisms.

## 5 Conclusion and Future Prospective

The use of polymeric nanoparticles in the biomedical and environmental fields is increasing daily. Once within the body, some metallic and polymeric NPs produce ROS causing damage to *Drosophila* in various ways. Multiple mechanisms are engaged in response to ROS. However, if the ROS levels become too high, they can cause damage to cells, tissues, organs, and the entire body. Several fly behavioral assays and phenotypic studies showed that the nanocomposite did not affect neural activity and phenotypes at highest and lowest concentrations. MnFe2O4@poly(*t*BGE-alt-PA) is appropriate for many biological applications since it does not cause any genotoxic and cytotoxic effects in *D. melanogaster*. The current study focuses on the *in vivo* toxicological analysis of green synthesized nanocomposite as a possible drug delivery mechanism and various environmental applications such as wastewater treatment and nanofertilizers. The toxicity assessment of the nanocomposite in *Drosophila* is found to be safe and nontoxic. It needs to be deliberated on other model organisms before the extensive use in drug-delivery systems in biomedical fields.

## Compliance with Ethics Requirements

This article does not contain any studies with human or animal subjects.

## Abbreviations

ROCOP: ring-opening copolymerization reaction
UV TGA: ultraviolet thermogravimetric analysis
DAPI202124: 4’,6-diamidino-2-phenylindole
ROS: reactive oxygen species
DCFH-DA: dichloro-dihydro-fluorescein diacetate
PBS: phosphate-buffered saline
NBT: nitroblue tetrazolium
GAA: glacial acetic acid
PFA: paraformaldehyde
μM: micro molar
μL mL: microliter millilitre
NOMPC: no mechanoreceptor potential C
CPG: central pattern generators
PNS: peripheral nervous system
s: seconds
SEM: scanning electron microscope
EDS: energy dispersive spectroscopy
DNA: deoxyribonucleic acid
